# Open Transparent Communication about Animals in Laboratories: Dialog for Multiple Voices and Multiple Audiences

**DOI:** 10.3390/ani11020368

**Published:** 2021-02-02

**Authors:** Larry Carbone

**Affiliations:** Independent Researcher, San Francisco, CA 94117, USA; larrycarbonedvm@gmail.com

**Keywords:** transparency, openness, communication, animal research

## Abstract

**Simple Summary:**

Over the past 10 years, animal research support groups in Europe have committed to greater openness and transparency of research institutions and scientists, a commitment that US labs could also take up. For openness initiatives to satisfy animal welfare advocates’ concerns, openness must be more than just showing more; it must invite feedback on what to show. In this article, I propose going further in the US, inviting animal welfare advocates into laboratories, onto ethics committees, and into any initiatives to update guidelines for animal care practices.

**Abstract:**

In this article, I offer insights and proposals to the current movement for increased openness and transparency about animal use in laboratories. Increased transparency cannot be total transparency—as no story or picture can ever be complete. When research advocates share their stories, they must decide which words and pictures to edit out. I ask here: Who of the listening “public” gets a chance to revisit this editing, and find the information that is important to them? To the extent that (what I call) the “new openness” attempts to speak to a “lay public” and exclude animal activists, I suggest that refinement-focused animal protectionists deserve enhanced avenues of openness and inclusion—which some research advocates might fear giving to more extreme activists and which a less invested “lay public” may not want or need. I conclude with some specific examples and suggestions to not just invite inquiry from animal advocates, but to bring them in as witnesses and participants, to learn from and incorporate their concerns, priorities, expertise, and suggestions. This can bring a diversity of ideas and values that could improve the quality of science, the credibility of animal researchers, and the welfare of the animals in laboratories.

## 1. Introduction

*Pangolin* is now a household word, thanks to the 2020s’ COVID pandemic. Bats have made headlines, as the likely reservoir of COVID and other zoonoses, itself another household word for 2020. Laboratory animals, too, have been in the news, as people track the progress of vaccines from cells to mice to monkeys to people. Newspapers report on these mice and monkeys, as well as the llamas, hamsters, ferrets, cats, dogs, and others that COVID research has conscripted.

What should people know about animals in COVID or other laboratories? What do they want to know? Why should anyone tell them about it? For over a century, scientists and their critics have argued over what access anyone should have to seeing or knowing how scientists use animals. In the past decade, with the UK’s *Concordat on Openness in Animal Research* at the forefront, some scientists and research institutions are trying to move past a “state of siege” to a greater friendlier invitation to the general public to “come see our [animal research] world” [1,2,3,4,5]. In what I call here the New Openness, they are moving the responsibility for defending animal research from support organizations to the individuals and institutions directly engaged in animal use. Perhaps these new initiatives of openness, coupled with initiatives for greater transparency in scientific manuscripts, will lead to a better public understanding of animal research, and the main goal of many of these efforts, greater public acceptance and support [6,7,8].

I see three main venues in the New Openness. All keep a “general public” audience in mind, but the giver of information may be at the institutional level as in the Concordat, at the individual scientist level (as well as scientific societies, such as the Society for Neuroscience), or at the support-staff and student level, including Biomedical Research Awareness Days that may be at an institution, but mostly the product of its grassroots [9,10,11,12,13]. In this essay, I put these recent developments into a broader and more historical context, focusing on the scene in the United States. This broader context ranges from scientists’ technical descriptions of their animal experiments to institutions’ required information-sharing, with inspectors, regulatory reports, and responses to open records requests for information. The historical perspective includes decades of scientists’ strategies for maintaining secrecy about animal use competing with outsiders’ strategies—some legal, others not—for obtaining researchers’ information. This competition that started in the days of paper-only communications continues in these days of electronic technologies, where both insiders and outsiders can broadcast descriptions and images, broadly or to targeted audiences. 

My perspective in this essay is United States-focused, but with an eye on developments in the UK, EU, and other countries, that Americans may learn from, adopt, emulate, or reject. I do not here offer a full catalog of openness and transparency actions. Most discussions of these efforts are anecdotal and live on websites, in news stories, or in descriptive articles in professional journals [4,14]. Empirical or critical analyses of these New Openness initiatives, or their effect on public understanding or support of animal use in research would be helpful, but are lacking. They could inform US considerations of whether to follow UK and EU examples. 

Some salient differences between the US and other Western countries influence efforts toward increased openness. EU requires far more extensive institutional reporting on animal use than the US, especially in the case of mice and rats that are not even defined as “animals” in the US Animal Welfare Act [15,16]. Thus, the standards of openness that the Concordat, the UK-based Speaking of Research, or the European Animal Research Association (EARA) endorse may be a smaller step in Europe than they would be in the US [2,16,17]. The UK also has two private charities, the Royal Society for the Prevention of Cruelty to Animals (RSPCA) and the Universities Federation for Animal Welfare (UFAW), who appear to have greater access to collaborate and communicate with the research community than comparable US organizations currently do [18,19]. 

Both the UK and the US have experienced assorted laboratory break-ins infiltrations, and exposes over many years. The UK also saw a level of animal activist violence and vandalism through the 1990s that was never quite matched, thankfully, in the US [4,20,21]. Nevertheless, I believe the threat of violence has always felt just as real in the US as elsewhere, and coupled with the aftermath of various non-violent activist actions, it has potently led researchers around the world toward keeping their heads down. 

Public opinion does matter. Leaked or stolen information has had major effects, and not just the targeting of individuals and institutions, but in regulation as well. In the United States, legally-conducted journalistic exposés of dog trafficking in the 1960s and laboratory infiltration and stolen video in two monkey labs in the 1980s led to the initial passage and subsequent major amendment of the Animal Welfare Act [22]. The Animal Welfare Act (AWA) mandates animal welfare standards, as well as some level of transparency, such as annual reports and government inspections, both of which appear on a government website. In the UK, infiltration at an animal breeder led to harassment of the staff to the point of graverobbing, the sort of outcomes opponents of the New Openness fear [23]. Public opinion can also work in scientists’ favor. In this case of harassment, news coverage alarmed the public and contributed to the passage of the Serious Organized Crime & Police Act, which may have largely ended animal rights extremist acts and created enough safety for openness initiatives like the Concordat [4]. 

What I call the New Openness with the Concordat at its leading edge, builds on this history. It is new in some important ways, reflecting two early 21st century realities: (1) Antivivisectionist violence, which was strongest in the UK, but cast a shadow over many other countries, has abated, at least for now and (2) current technologies allow the widespread circulation of information, claims, videos, and communications. Whereas, past efforts to promote animal use in science were largely individuals’ efforts, with permission if not encouragement of their home institutions, the Concordat is a commitment at the institutional level, currently with virtually every animal lab and animal research funder in the UK on board, and with similar transparency agreements in Spain, Portugal and Belgium [5,24]. And going beyond sporadic press releases, lectures, and interviews of yore, the Concordat’s main tools are its members’ and its own websites: Proactively displaying information about animal use in a way that anyone at any time can log on, view, and pass along to one’s own online community. Specifically, Concordat member organizations sign on to four commitments:

- We will be clear about how, when, and why we use animals in research;
- We will enhance our communication with the media and the public about our research using animals;
- We will be proactive in providing opportunities for the public to find out about research using animals;
- We will report our progress annually and share our experiences [2].

Consider the varied who, what, when, where, and why of animal research communications in the new openness. 

*Who*: Openness or transparency can mean different things to the research insiders, the animal protectionist outsiders, and the “general public” in the “troubled middle” that they both are vying to influence [25]. 

*Where and when*: Some communications reside on websites, viewable day or night; some require scheduled on-site interactions, inspections, or visits; some are texts freely given (manuscripts) or less freely (responses to public records requests. 

*Why*: Motivators vary from countering outsiders’ (mis)information to make for a better-informed public, to complying with mandated reports and inspections, to hopes of convincing an open-minded, concerned public to be supportive in some small or large way of laboratory animal use to feeling a moral obligation to account for the privileges (and funds) that society gives them [12,14,26,27,28,29]. If institutions and individuals put greater effort into showing and telling more than they used to, is this primarily self-interest, an “instrumental action” (Habermas’ term) to *convince* others of the value of animal research? Or more a sense of moral obligation, a “communicative action” to *answer* the questions and concerns and interests of research outsiders, seeking agreement rather than persuasion [1,30]? Institutions may take basically four approaches in their public-facing websites:

1. Defensive: State that they use animals, but that they follow all laws and maintain the highest standards of animal welfare. This is a common approach in the US.
2. Advocacy: State that they use animals, with pictures and case studies showing the value of animal research. This is how I see the Speaking of Research and EARA approach [16,17]
3. Transparent openness: State that they use animals, with pictures and case studies showing the value of animal research as well as statements on the harms to animals and limitations of animal research, so that readers can make their own informed judgements. This is the commitment of the UK Concordat and in European openness agreements [2,15].
4. Invitational openness: Going beyond the Concordat and current openness agreements, institutions can pro-actively invite questions, comments, visits and even participation, such as ethics committee membership.

*What*: My goal in this essay is to sort out what increased, but still partial, information insiders release in the name of openness, what they hold back, and who gets a voice on making that determination. 

## 2. Full Transparency and Complete Openness Are Not Possible

No one should take calls or claims of “full transparency” and be “completely open” literally, no matter what animal advocates want or scientists may promise [5,31,32,33,34,35,36,37,38,39]. 

An exercise to illustrate this point: Try writing an email to an interested friend about your last meeting with your supervisor or professor. Your friend is breathless; they want to know *every*thing. Now, what did you tell? Who said what, certainly, though likely not a literal transcript of a lengthy meeting. Did you include details of where you met, who got there first, what you each wore, what you ate if there was food? Were you both taking notes, with a pen, pencil or computer? Using your left hand or right hand? What did you want to say, but never got to? No, it would be impossible to describe everything about a meeting, or a series of experiments, or about the animals in your facility. You offer a mix of the things you find most important and interesting and what you believe your reader will find interesting. Your complete communication is, in truth, a partial account. Knowing this is not the full and complete story, your friend could become suspicious. What did you edit out, and why? Maybe the edited parts are, in fact, the most important to your friend. Do you give them a chance to ask for those details?

As with speaking and writing, so too with showing. You may throw your curtains open and tell me to look inside, but we both know there are corners of the room I cannot see. I may be most curious about what’s under the table or in the cupboards, but my view through your fully transparent window does not allow me to see. If you alone set the agenda for this complete, open, transparent view into your life, I will always wonder just what you were hiding. If we have had a century of mutual distrust, as animal researchers and animal activists do, I will assume the worst, that this is showmanship rather than invitational openness. 

In the same way, information about animal use will always be partial. A one-way email message, like a scientific manuscript, press release, television show, or a lab’s web page, may be informative, but if it is not invitational, inviting questions and even comments, does it count as openness [9,40]? Messaging is not dialogue. Marketing is not dialogue. Through open dialogue, scientists and their supporters allow listeners, including critical, skeptical listeners, a role in setting the agenda of what information they receive. Calls for “full” or “complete” openness, transparency, and information-sharing miss the mark if they are solely about one-way communications that laboratories emit. 

One-way communication always entails some degree of editing or curating, even when the communicator is eager to teach, inform or show their listener all that the communicator thinks is interesting and important. This is not necessarily nefarious, unless someone knowingly poses as presenting the whole truth and nothing but the truth when they know they cannot and are not doing that. 

True openness requires avenues for activists, journalists, skeptical outsiders, even just high-school students to see or hear about the animals to get the information that *they* identify as important to *them*. Tours, classroom visits, and some innovative media have been staples of openness efforts through the years; the Concordat encourages limited facility access to media and public, but does not require it [2]. Live interactions (of course, live via the internet in the era of COVID) offer a greater chance for information-receivers to ask the questions that are important to them, or to see things that they prioritize [40,41,42]. Frequently, in my experience giving tours, classroom presentations, or otherwise responding to outsiders’ questions, we reach a limit of what I feel I can show or tell. My audience may control the questions, but I still control the answers. The challenging discussions are not about whether to share information, nor about how much to share, but about who determines what information animal users will share and what will be kept back. 

My own work history affects how I read the decades of debates about openness and secrecy. Until 2019, I was a laboratory animal veterinarian and ethics committee administrator, in academia. My role included interaction with Animal Welfare Act AWA government inspectors, advising media staff on press releases following AWA citations, reporting animal welfare issues to the National Institutes of Health (NIH) Office of Laboratory Animal Welfare (OLAW). I have always resisted any call to be a university spokesman on animal issues, stating I’d rather work behind the scenes to make a quality animal care program others could proudly represent. I have seen my name on but a single animal rights pamphlet as the man to call with complaints about the university’s animal use. Though I received one threatening phone message some 20 years ago, I have certainly not been targeted, and in fact, have been invited to various meetings bringing together research insiders with activists and animal liberation academics. Madhusree Mukerjee reviewed my 2004 book on the history of the US Animal Welfare Act, noting that I gave “few peeks behind the door” of the animal lab, with a description of my life in the lab that was “distant, measured, and worded with sometimes excruciating care [22,43].” My generation learned to carefully guard information about the animals, writing medical records or answering public records requests with a goal of giving nothing to activists that could be used against the university. 

More than most animal research insiders, I also conduct animal welfare policy research, and in the process, I become an outsider looking in at others’ animal use practices. A recent project required learning to use the AWA website of facilities’ inspection findings and annual reports, and how to craft public records requests to NIH OLAW and to public universities [44,45,46]. My project required comparing publicly accessible information on primate, dog, and other AWA-covered species at publicly funded laboratories with their statistics on annual mouse and rat use, which is not information available from the government [47]. Most universities are exempt from public records laws, as are private pharmaceutical companies, so I supplemented public records requests with individual requests to private universities, asking them to voluntarily release their mouse and rat use statistics, reminding them that their other species’ data were publicly accessible. Only five of 20 released their rodent-use data. 

My personal story well illustrates differences between the US and European systems, with their contrasting approaches to regulatory oversight and data availability [2,4,5,40]. Thus, in this essay, I look to other countries’ developments as possible harbingers of changes that may come to the United States. 

## 3. Who: Insiders, Outsiders, and the “General Public”

The *Concordat on Openness in Animal Research* commits its member institutions to “be as open as possible in sharing information with the public”, with commitments to provide information, as well as to respond to “reasonable enquiries” [2]. The Concordat’s framers recognized what has been true for a long time: That “the public” outside the halls of scientific research is a varied lot with a range of views on scientists’ use of animals, and a range of commitments to acting on those views [48,49,50]. 

There are many overlapping communities in discussions of animal use ethics and communications, and people use different terms. Even avoiding the most obviously pejorative, such as crazies and vivisectors, torturers and zealots, language matters and can subtly foster prejudices about “the other side”. For this essay, some words I choose, hoping that any readers can see which category(ies) they belong to, and feel that label respectfully reflects their identity:

Insiders: People with first-hand knowledge of animal laboratories, including scientists, animal care support staff, veterinarians, research administrators, ethics committee members, the staff at research defense societies. 

Outsiders: Everyone else, including students, professors, and staff at universities and research companies who have no involvement with animal research

Animal welfare advocate: Either an outsider or insider with a strong commitment to improving animals’ welfare. I do not use this term to distinguish people who are permissive of animal use from those with a more abolitionist stance. 

Animal activist: In this essay, outsiders whose agenda tacks toward shutting down animal research. Most activists use non-violent, legal means, but I include here also people who use threats, violence, vandalism, infiltration, or illegal acts.

Animal protectionist: A very broad term I use for outsiders who are in any way actively engaged in promoting animal interests, ranging from philosophers, the staff of prevention-of-cruelty organizations to more strident or radical activists. 

Research advocate: Scientists, veterinarians, lobbyists, administrators, educators, and others tasked with assuring that animal research will continue. 

Regulators: Government officials whose role is oversight of animal research laboratories, as well as politicians who may occasionally consider legislation to change levels of funding or levels of oversight of animal research.

The Public: Basically, none of the above. For present purposes, the public includes anyone not actively involved in animal research issues, even if they hold membership in an organization that does some research advocacy or funding (such as the American Medical Association or the March of Dimes) or an animal protection organization (such as People for the Ethical Treatment of Animals (PETA) or a local humane shelter). Most journalists are the Public if they are writing about, instead of writing for or against, animals in laboratories.

**Figure 1:**
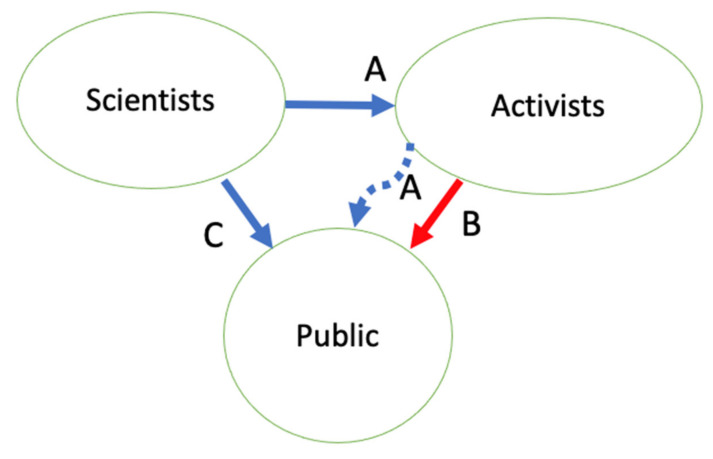
Animal-use information flow, as perceived by some research defenders. (**A**) Activists take information from science and scientists and mischaracterize it to pass on to the public. (**B**) Activists disseminate information with no basis. (**C**) Scientists bypass communication with activists to directly show and tell the public their version of laboratory animal use.

**Figure 2:**
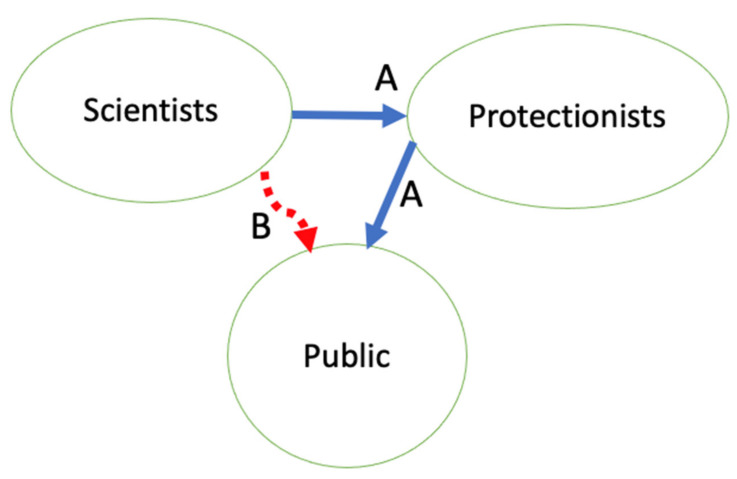
Animal-use information flow, as perceived by some protectionists. (**A**) Protectionists obtain information from science and scientists and to pass on to the public. (**B**) Scientists bypass communication with protectionists and release partial and misleading information about animals.

At its simplest, there are two sets of outsiders, the animal protectionists, and the lay public. People in the animal laboratories and their supporters vie with activists outside the institution to tell “the public” about animal research. Research advocates accuse critics of distorting the information they get from laboratories, and of fabricating claims to sway this public to their beliefs (information flows A and B in Figure 1) [4,51,52]. The New Openness is public-focused (C in Figure 1), though with no illusion that activists are not also watching and listening. For their part, protectionists see a situation (Figure 2), in which researchers hide or misrepresent their animal research, leaving the press and public reliant on protectionist outsiders to obtain the full story and pass it on [14,39].

The reality is much more complicated than three non-overlapping communities of Protectionists, Scientists, and Public, and communicators and audience do not map precisely onto the categories of insider and outsider. Table 1 lists many insiders who may communicate information about animal use, along with the outsiders (activists, journalists, government inspectors) who pass along their understanding of what they’ve seen or heard from the laboratories. These varied communicators reach a wide range of audiences, including some, such as students and non-science staff, within the institution (Table 2).

At contest are the sympathies of a presumed “general public” that wants medical progress, responsible use of public monies, and humane treatment of animals, and knows little of the intricacies of animal research, or of scientific research, in general [25,53]. At contest, too, are the financial and government decision-makers this public might influence. Vying for public support and government action are various pro-animal use groups and individuals arrayed against assorted animal protectionists; these are not monoliths, and many within these two camps share an interest in working together on policies that might improve animal welfare and allow continued animal use. 

Though Figure 1 and Figure 2 depict Public and Activists/Protectionists as non-overlapping sets, each captured with a single-word label, both contain a broad spectrum, and the two sets do overlap. “The public” includes negligent pet owners, vegan donors to PETA, veterinarians, and physicians in community practices, and millions of people who never give much thought to animals, in general, or in laboratories.

For this essay and as my general practice, I use the word “Protectionist” as it covers a much broader range of people actively working on behalf of animals than do words like activist, antivivisectionist, welfarist, watchdog, advocate, liberationist, or “the humane community”. The range is broad, and occasionally, it is necessary to distinguish among the various overlapping protectionist groups and individuals. The vocal and visible come to mind, not just the more extreme people who may use threats, vandalism, infiltrations, but also people whose occasional attendance at a demonstration or donation to PETA marks them as sympathizers of antivivisectionism. Shelter workers work mightily to protect the dogs and cats they care for, but will still have a range of opinions on animals in medical research. Among the professional staff of various animal welfare and animal rights organizations, some may seek access to information with a goal of replacing, reducing, and refining as much animal use as possible as quickly as possible. The RSPCA and the Universities Federation for Animal Welfare, both in the UK, seem to come closer to the access necessary for this role than most organizations [18,19]. Exclusion of welfare advocacy groups is more the norm in the US in my experience.

But where do animal protectionists fit in the new openness initiatives? Are they people with whom research advocates should engage, or should they be bypassed, ignored, or refuted *in absentia* when animal researchers talk to the (non-protectionist) public [51,53]? Is it even possible to have an open and transparent discussion with “the public”, while excluding this segment of the public? Are they best left in the third person, talked *about* as the spreaders of misinformation? Or can research advocates give them a chance to bring their concerns into the laboratories?

## 4. Animal Protectionists, the Status Quo Secrecy, and the New Openness

Animal scientists face accusations of secrecy, but secrecy can be leaky; word gets out.

A challenge with secrecy is that while the “general public” may get little insight into a facility’s animal use, committed activists and watchdogs know the tricks for getting information. Where I worked, we denied journalists’ requests for visits, put out no pro-active press releases on issues like renewed accreditation or revitalized animal facilities, and met few of Speaking of Research or EARA criteria for grading research institutions’ public-facing websites [16,17]. Despite our attempts at a low profile, activists knew how to file Public Records Act requests we had to answer, how to submit Freedom of Information requests for our file at the NIH, how to obtain our AWA inspection reports, and how to publicize unattractive information they found.

In a digital age, it is simple to conduct a literature search with the name of a nearby institution and words like “primate” or “animal model” to learn what research is being published in a person’s neighborhood. Long before PubMed or other search tools made that possible, scientists wrote manuscripts for a scientist audience, but they knew that others were watching too. Lederer describes how the Council on the Defense of Medical Research and editors at the *Journal of Experimental Medicine* mid-20th century scanned manuscripts for words antivivisectionists might seize on to scientists’ dismay [54]. They warned that antivivisectionists read manuscripts and score a failure to mention anesthetics as a failure to use anesthetics (a persistent problem today) [55]. They banned descriptions of animals vocalizing, moaning, or whining “as though the animal was in pain.” Photos were tightly edited to show just a body part, not the whole animal. Through the decades, dogs became “preparations”, and mice are “animal models.” Scientists can touch monkeys’ brain neurons, but somehow the words *surgery* or *analgesia* do not appear in their Methods [55]. 

A century later, the National Centre for the 3Rs (NC3Rs) launched its ARRIVE guidelines (Animal Research Reporting In Vivo Experiments) for increased and more transparent reporting of animals in scientific manuscripts (as well as the potential to improve laboratory animal welfare)) [6,7,8,56,57,58,59,60,61,62]. The ARRIVE guidelines aim to “ensure that studies are reported in enough detail to add to the knowledge base” [61]. They are a response to a concern that scientists themselves often do not understand how much information to provide to allow full evaluation or replication of their published work, not that they would intentionally obscure the information [56]. ARRIVE guidelines would certainly require clarity when monkeys have undergone surgery, with information on how scientists, veterinarians, and animal carers provided for their health, pain management, and welfare.

ARRIVE faces the same challenges of all descriptive writing: “Fully” reported descriptions are impossible [38]. The checklist contains far too many items (I count close to 40, and most of these are complex, with numerous subitems) to fit into any one manuscript. Scientists and journals still need to edit down to the partial information that seems most important for scientific critique or replication. For example, item #8a, *Experimental Animals*, includes species, sex, strain, age/developmental stage, and weight. Not only might some of those have minor relevance for certain studies, but some unlisted factors, such as litter size, number of females rearing the litter, which strain’s mitochondrial DNA a hybrid animal carries, or the microbiome profiles of the animals, might be highly salient for some other studies.

Authors and editors fully committed to transparency and diligently using the ARRIVE checklist, thus nevertheless publish edited (i.e., partial) descriptions of their research, even when online publishing makes possible fuller descriptions than previously possible. Moreover, items selected for their ability to scrutinize the results and replication of methods may not answer protectionists or welfare researchers’ questions about a project [55,58,59,60,61,62,63,64]. I propose that greater openness would require publishing an author’s ARRIVE checklist as a supplement to the main paper, plus a user-friendly way for readers to request the welfare-relevant information that is important to them to know.

As more and more journals, authors, reviewers, and editors employ the ARRIVE checklists, they will produce more complete and robust literature. This can only help scientists avoid unnecessary duplication, refine their own methods, and perform necessary replication with a goal of better science and better animal welfare. As I presented this work at a recent lecture, a young scientist reminded me of an unfortunate current limit to open science communication. A literature search may yield dozens of promising citations and abstracts a scientist should want to review in planning their project, but many of those will be behind a pay-for-access wall. It’s one thing to take the time and effort to obtain a few of the most important articles, but to do this for dozens of articles that may not have the value their title promised, is a significant barrier. Thus, the next frontier as ARRIVE raises the quality of reporting in animal research is assuring that scientists and others have reasonable access to this improving and important literature.

## 5. Toward Invitational Openness, in the US Context: Some Proposals

Though I know my own previous institution’s history in some detail, I see no reason to think that that university is any more or less guarded or non-transparent about its animal use than most US universities and companies. The US, in general, is moving more slowly toward the increased openness that the Concordat, the Basel Declaration (now renamed Animal Research Tomorrow), Speaking of Research, the EARA or other countries’ openness initiatives are modeling [4,5,26,40,65,66,67]. I am not alone in my dual belief that animal research can produce vital knowledge for promoting human, animal, and environmental health and that sentient animals deserve stronger welfare protections in laboratories.

I have said here that complete or full openness and transparency are mythological creatures; all stories and shows are partial, edited, curated depictions of bigger truths. I have shown that secrecy is also partial and leaky, leading to the communication flows in Figure 1 and Figure 2 in which protectionists procure information however they can, then pass on this partial information to the “general public” with whatever spin they choose to put on it. I’ve reminded readers that the catch-all term Protectionists includes those activists that science institutions may fear, but also knowledgeable, committed, reformist animal welfare experts they could learn to welcome, engage, trust, and listen to.

Now I want to look at some past and present versions of invitational openness, some in the US, and challenge the research community to pursue this collegial possibility. Some of these suggestions could be easy, some will meet quick resistance, and one would require an act of Congress. This long list faces an illustrative deficit: My own limited imagination after so many years as an animal research insider. Part of the value of inclusiveness is that the animal advocates currently looking for more opportunities to comment and contribute will bring their ideas to the table and expand the vision of collaborative communication.

### 5.1. Inviting Protectionist Input on Developing Openness Initiatives

In its preparation to develop the Concordat, the organizers at Understanding Animal Research invited research outsiders to answer poll questions and join working groups to frame a proposal for openness that addresses concerns of what interested outsiders want to know. Protectionists want access to insider information for their own use, and also want a voice in framing what researchers will tell the more general public. The protectionist group, RSPCA, joined discussions, and the RSPCA remains involved. The British Union for the Abolition of Vivisection (BUAV, now the Cruelty Free International Trust) submitted a video while declining more direct participation [39]. I saw a similar pattern in reviewing public comments while studying the history of the US AWA amendments. Law requires a public comment period, and in the 1980s, abolitionist groups sometimes stayed apart while advocacy groups, such as the Animal Legal Defense Fund, the Humane Society of the United States, and especially the Animal Welfare Institute, took the opportunity to make concrete proposals for reform [22]. This sort of invitation for input, whether voluntary (UAR) or required (AWA), allows outside groups and individuals some control of the level and type of engagement they want.

I draw a distinction between these invitations for input versus the types of public surveys that can show what a general public might care about to market a message that will persuade, convince or garner support [53,68,69]. The distinction may be subtle, and requesting public input may serve more than one goal. Sandgren, a medical research scientist who has worked to communicate with the local animal protection community in Madison, Wisconsin, tries to straddle the distinction between strategic listening with a goal of persuasion and meeting an obligation to inform outsiders about what they want to know more about. He writes: “Through my interactions with animal activists, I’ve discovered that we share several beliefs. In particular, we each feel that the public will agree with ‘our side’ if they have all the facts [27]”.

### 5.2. Mice and Rats Need to Legally Become Research Animals in the US

Regulatory oversight is an important component of laboratory animal welfare, and part of that in most jurisdictions includes openly available self-reports of compliance and of annual performance metrics. The UK Concordat calls on its members to post these reports for all to see, and the University of Oxford is one example with a very informative website, even listing the number of primates on “severe” research projects [70]. By contrast, the patchwork system in the US results in entire businesses or colleges flying beneath the radar as the US AWA does not count rats and mice as “animals”, and the NIH does not count them as “animals” unless they are federally-funded research projects [47]. In the age of the new openness, they are hidden. Only the US Congress can complete rodents’ evolution into countable animals, which currently seems unlikely. If nothing else, the NIH could standardize how they expect institutions to report their animal counts, and it could aggregate those numbers for easy, transparent public viewing. More, making mice “animals” would allow government veterinarian inspectors to see them and evaluate their care, with inspection reports available for the public to see.

### 5.3. Include Descriptions of Harms and Limitations, and Invitational Openness as Items for Grading Institutional Websites

Speaking of Research and the EARA have grading systems for institutions that post their Animal Research Statements and related information online, based somewhat on the four commitments of the UK Concordat [16,17,48]. The Concordat is explicit that its commitment to clarity about how and why people use animals includes discussing not just the benefits but also the harms and limitations of animal research. Discussion of harms and limitations is not part of the Speaking of Research or EARA grading systems; it should be. I would also tie such a scoring system to any quasi-binding concordats that universities and companies sign onto, with a fifth element: Clarity on how any person can easily make quick contact with questions, requests, or comments. Set acceptable-use guidelines as online magazines and blogs do, to filter out abusive comments, but let “the public”, as well as protectionists comment on the institution’s website.

### 5.4. Openly Sharing the Animal Welfare Costs in Research: Known Harms Plus Non-Compliances

Bad things happen to animals in laboratories. Mostly, this is because some experiments will cause pain or distress or confinement or death. Occasionally, scientists, veterinarians, caregivers, or others may knowingly or unknowingly fail at their commitments to the animals. What should the public know about the incidence and causes of these animal harms?

The UK Concordat commits organizations to “provide accurate descriptions of the benefits, harms and limitations” of their research [2]. I do not currently see a comparable US commitment to openly discussing potential harms and limitations. The UK Home Office aggregates institutions’ lay summaries of animal project licenses, allowing outsiders to make their own assessment of benefit to harm [71,72]. Quite the opposite in the US: Current advocacy campaigns, such as *Come See our World, Biomedical Research Awareness Day*, or *Love Animals? Support Animal Research* make little mention of animal harms, instead assuring a concerned public that tough regulations and ethics committees are there for the animals [3,11,72]. A child writing a class paper, for example, can read the competing realities of the Foundation for Biomedical Research (FBR) or of PETA and find little reason to believe or trust one version versus the other, or know that there are scientists and protectionists seeking some common ground beyond these binaries.

We faced a related issue while updating my university’s animal ethics committee web site. As we developed illustrated descriptors for researchers on the committee-endorsed ways to do various procedures (e.g., blood collection from mice) we sampled other universities’ web sites to make sure that our recommendations were within industry standards. And we noticed in the process that more and more campuses seemed to be moving such information behind password-protected firewalls, fearful that activists would find those sites and use them negatively. I credit our Chief Ethics and Compliance officer at the time for appreciating that secretiveness would diminish universities’ ability to stay informed on best practices, and if our example would help others promote best practices, even for procedures that would harm or kill animals, we had an obligation to keep our site available.

Reed has argued that this is exactly the fuller information the public would need to make an informed decision on the costs and benefits of animal use [73]. It might promote efforts to identify issues in housing and experimental design in need of refinement. In the UK, the Concordat, which has invited and included RSPCA participation, has recognized a need to communicate the harms to laboratory animals, and in its annual self-review, has identified a need for more of its signatories to do more on this front [4,48,74]. In the US, the window into harmful animal protocols is via AWA annual reports, posted on the USDA website, listing every institution’s use of AWA-covered animals in 3 “pain categories,” along with explanations for withholding pain medications for Category E animals. Thus, dedicated activists with an anti-research agenda know how to find this information, but few if any US institutions proactively provide the general public information on the numbers of animals they use or the pain or severity classifications of those uses [47].

Alongside the expected harms of approved projects, animals may suffer from various non-compliances. The US and UK government agencies both post compliance information, whether Animal Welfare Act violations in the US or Home Office investigations [44,75]. In the US, the AWA covers but a fraction of animals in laboratories. Animal protectionists can use public records requests to obtain self-reports to the NIH (for animals on federally funded research). Certainly, various protectionist groups want access to this information, whether they be activists hoping to galvanize public outrage, or reformers wanting to know the scope of the problem of research non-compliance. This sort of information might also help the research community target improved training and oversight efforts, with some public transparency about those efforts. The University of Wisconsin-Madison is that rare example of a US institution that proactively gives the public the information on its AWA compliance that activists know how to easily find online [76].

Animals do experience harm in laboratories, and the people an institution might least want to know about them already know the tools to find self-reports of non-compliance, annual reports of animal use in the various pain categories, and government inspection reports. The UK Concordat requires discussion of potential harms to animals (though not of non-compliances). I suggest that Speaking of Research and EARA include transparency about harms and non-compliance in their grading systems for animal user websites, and that all institutions truly interested in openness pro-actively share compliance and animal-use statistics.

### 5.5. Video Tours with 21st Century Technology

Technology advances have allowed willing organizations to offer video tours of their animals. Viewers can see the monkeys in their outdoor corrals at the Oregon National Regional Primate Center [42]. They can see healthy pigs on an African Swine Fever project rooting around or interacting with the staff. In a move toward transparency, they even include images of pigs succumbing to the disease in the laboratory [77]. They can even watch macaque monkeys with metal implants attached to their skulls in an Oxford University lab, the university whose animal activist protests and threats spawned student movements in support of research [4,51]. These are excellent efforts. I particularly encourage viewers to watch the Oxford monkeys closely for any evidence that metal devices attached to their heads are bothering them as they move about; I see no such evidence [78]. You will not, in a virtual or on-site tour, get access to the Oregon monkeys “on study” in the labs, or see the totality of an Oxford monkey’s day. The Oregon and Oxford videos show so much more than scientists have been willing to show in the past, but they are still carefully edited versions of the full picture. A common and true claim for why lab tours cannot show more is that tours pose infectious disease concerns, interruptions of data-collection, and stress to the animals. This can even be true of lab visits by the ethics committee or the campus veterinarians. A suggestion I expect no research institution will take me up on: Install live cams in the areas you know that outsiders will be most interested in seeing, with the same around-the-room view in the pre-recorded Oxford videos. Researchers already use these so they can monitor their animals without entering the room; it would be simple to set them up as their own publicly-viewable video stream.

It is true that activists will use these videos in their anti-animal-use campaigns, as they presently use video they can obtain [79]. Research advocates claim that activists use outdated images that do not show modern refined animal care; perhaps it is better that activists show current images. I could see also using this as a chance to educate outsiders, for example linking to a short tutorial on how an animal welfare scientist would evaluate a monkey’s reaction to going into a restrainer, performing tasks, responding to researchers entering the room.

### 5.6. Animal Protectionists on Ethics Committees

A full discussion of the function and alternative proposals of animal ethics committees is too lengthy for this essay; my focus is on ethics committee membership as it relates to issues of openness. In the US, most research institutions are required to have an ethics committee (typically called an IACUC, i.e., Institutional Animal Care and Use Committee). Those that operate under AWA rules (because of the species they use) or NIH rules (because of their funding stream) or for any other reason adhering to the *Guide for the Care and Use of Laboratory Animals* (the *Guide*), must have a “public member” and a non-scientist, though one person may fill both roles. This is a powerful role, as committee members go beyond whatever information an institution posts, joining in on semi-annual self-inspections and review of protocol compliance. They have a voice in evaluating how well the objectives of the study justify potential animal welfare concerns. Along with scientists, veterinarians, and other insiders, they are not just watching, but actively shaping the lives of the animals in the laboratories.

When one individual is both the only non-scientist and the only public member of a committee, the potential for intimidation is high. The US could adopt practices some other countries use, such as the proportional representation of community members rather than “at least one”, as well as criteria for who chooses community members [80,81,82]. In particular, I would like to see more US institutions working with local or national humane and animal welfare organizations to recruit ethics committee members, and giving those members security and tenure that the institution can only override with clear, written reasons.

Expanded numbers and categories of “unaffiliated’ or public ethics committee members would continue to bring the fresh eyes of true novice non-scientist members. Done correctly, science-trained animal welfare advocates could contribute expertise, but from the protectionist perspective. Is yet another certification in laboratory animal science really necessary? Perhaps not, but I could see groups, such as Public Responsibility in Research and Medicine (PRIM&R), the Humane Society of the United States, the Animal Welfare Institute, as well as academic programs in animal welfare science, producing a cadre of knowledgeable welfare specialists who would fill these roles. This proposal moves openness from the expanded posted information that characterizes Concordat membership to the right for open access to information and an obligation to work with the institutions’ veterinarians and scientists to co-produce best practices.

### 5.7. Animal Research Insiders and Outsiders Can Increasingly Co-Produce Animal Welfare Practices and Standards

Animal advocates and research insiders have worked for years to co-produce best practices for welfare-minded improvements in laboratory animal care and use. Before the Animal Care Panel (now the American Association for Laboratory Animal Science, or AALAS) wrote the first *Guide* in 1963, the Animal Welfare Institute (AWI) enlisted scientists, veterinarians, and others to write its 1953 *Basic Care of Experimental Animals* (now *Comfortable Quarters of Laboratory Animals*, in its 10th edition) [83,84,85]. Then, as now, the AWI invited the expertise of laboratory insiders, particularly veterinarians and animal welfare scientists, to promote evidence-based suggestions to maximize welfare in housing animals for research. Animal protectionists have worked with science insiders on a number of issues, co-producing documents and hosting assorted conferences to bring together interested parties in search of common ground to benefit animals [86,87]. In the 1990s, the National Antivivisection Society sued NIH to ban the use of the mouse ascites method to produce monoclonal antibodies. In response, the National Academy of Sciences included a representative of the Humane Society of the United States onto a panel with scientists and veterinarians to review possible replacements and refinements; that use of mice is now mostly obsolete [88,89]. Other such joint ventures have covered sepsis models, use of carbon dioxide for rodent euthanasia, laboratory animal distress, welfare assessment, dogs in toxicity testing, use of dogs in federally-funded research, housing animals in Danish laboratories, and much more [90,91,92,93,94,95,96]. Again, these collaborations go beyond showing outsiders that researchers care about animal welfare to inviting them in (or accepting their invitations), for collaboration to produce knowledge and standards for animal welfare [97,98,99].

Animal welfare standards should combine the best available facts with sound values and ethics, though it can be easy for those of us rooted in sciences to think we can move from animal welfare data to animal welfare standards in an objective value-neutral way, continuing an ideology that science and data can exist in a value-free vacuum [97,98]. I suggest that conferences on animal welfare and laboratory animal science always include animal protectionists in their planning and their programs, and certainly as attendees; for some, this might be a move closer to their original practices, a return to more optimistic, less polarized times. And yes, I would invite expert protectionists to work on seemingly technical or veterinary documents, like the *Guide*, pain management standards for primates, the American Veterinary Medical Association guidelines on animal euthanasia, the National Academies guidelines for animals in neuroscience research, or new programs to promote the 3Rs in the US [99,100,101,102,103]. None of these are narrowly technical documents, but rather efforts at applied animal welfare, and I would encourage the broadest possible expertise and diversity of opinion in their production.

### 5.8. Public Records Requests

I have no solution to offer, but let me define a problem I see, having worked both to respond to records requests and to obtain information via that route. In the face of closed doors and drawn curtains, outsiders use the tool of various Freedom of Information (FoI) and Public Records Act requests, while institutions hone their skills at limiting what they release. Activists can weaponize FoI requests, not just scouring for information to turn into targeted campaigns, but also to force institutions to devote resources to meeting FoI demands for documents [104]. Institutions develop practices and infrastructure to limit FoI responses to what is strictly required of them, with government guidance and cooperation on keeping communications under the FoI radar (e.g., via telephone) [46]. I have seen no data on how often the big exposés and campaigns have relied on FoI information, as opposed to various leaks, infiltrations, whistleblower actions, or government inspections. Is it possible the threat of FoI releases does not warrant all the efforts in making and responding to them? As an insider acting on behalf of the university administration, I spent so many hours searching for records and redacting them (though the requestors are likely quite satisfied to receive unredacted records) to respond to FoI requests. Under common FoI rules, the obligation is to release records, not to generate records or to answer simple requests. When activists were working on campaigns to mandate laboratory cat and dog adoptions, we searched through records documenting the “disposition” of those animals, when I could easily have responded in 2 paragraphs describing our adoption practices and the approximate numbers of adopted versus euthanized animals. As an outsider looking in, with a concern that mouse use in the US gets insufficient regulatory oversight, I’ve been concerned not to unfairly downplay whatever site visits the NIH OLAW conducts in mouse laboratories, partly making up for the lack of AWA inclusion of mice. That simple question, “About how many site visits does OLAW conduct in a typical pre-COVID year?” could be quite simple for that small office to answer in a 1-sentence email. But OLAW does not have in its office a document tallying a year’s site visits, and so instead, I waited three months via the FoI process to get copies of OLAW site visit correspondence, containing far more information than I need, to count the 10 or so site visits per year myself. It was such a waste of their time and mine.

The EU Directive 2010/63/EU calls on member states to keep the public informed by pro-actively posting anonymized non-scientific summaries of animal projects and annual statistics on animal use [15]. The current system in the US is reactive, via FoI processes, with neither the *Guide*, the AWA, or the NIH rules, and promotes a reflexive response to inquiries that institutions will only release the information they are legally bound to release. Current FoI practices do not promote any of the goals of the New Openness and work against trust-building. To tell someone you have the information they want, which you both know you are obligated to release, but that you will only share that info via a formal, lengthy, and laborious FoI process is not the invitational openness we should be striving for.

### 5.9. Reciprocity: What Do Research Advocates Want from Animal Advocates?

I cannot presume to speak for the research community, which is hardly a monolith. Many scientists, veterinarians, and technicians are themselves discomfited by the animal harms their research entails, and might welcome challenging questions and suggestions about their work. They want something other than the “my side versus the other side” engagement [1,105]. They want to be able to talk to people they meet about the work they do, both the good and the bad, without fearing vilification [106,107].

Protectionist organizations vary. I do not see the various Frequently Asked Questions about animal research information at many animal advocacy organizations to be misleading or accusatory, and they can serve as an exemplar for others [18,19,108,109]. It is fine that protectionists organizations express their desire that someday animal research is no longer seen as necessary; many scientists working with animals feel the same way, even if they disagree on how soon that day may come. As long as a website talks about the 3Rs alternatives (those three Rs being the reduction, refinement, and replacement efforts that Russell and Burch elaborated in the 1950s), I take them as open to dialogue with scientists [110]. I want protectionists to do fact-checks with knowledgeable insiders when necessary (and get answers!), then do their best to honestly represent what researchers have told them, however much they may disagree. I want them to continue joining in activities, such as the World Congress on Alternatives, where insiders and outsiders seek common ground for animal welfare, and hope that more and more such meetings will open or reopen to them [86]. I encourage animal advocates, including institutional insiders, such as humanities faculty or other institutional staff, to seek positions on local animal ethics committees.

## 6. Conclusions

The New Openness of the Concordat and related efforts are currently mostly an effort to show more and tell more of life inside animal laboratories than ever shown in days gone by. Motivators may be primarily self-interest, to *convince* others of the value of animal research, or *obligational*, to answer the questions and concerns and interests of research outsiders, or a blend of the two. Any attempt to show or tell others what you know can only offer a partial story; every story is an edited version of the truth. If the goal is to truly meet a moral obligation of accountability, not only will the editing frame shift, but science insiders will invite outsiders, including animal advocates critical of the enterprise, to join in the editing process, ask questions, determine for themselves what information they want more of, and beyond that, to collaborate in being part of the story themselves.

**Figure 3:**
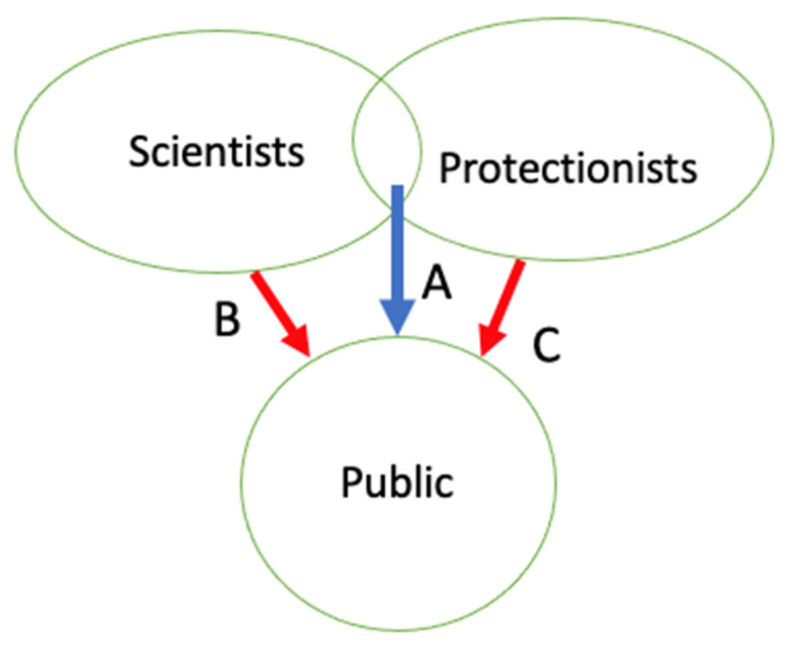
Animal-use information flow when scientists and animal protectionists work together to develop practices and knowledge—together sharing information with the public. (**A**) Protectionists and scientists co-produce knowledge and share it with the public with maximum opportunity for public input on what they want to know. (**B**,**C**) Scientists and protectionists maintain their right to honestly communicate their “side” where they do not reach a common understanding.

Inspired by efforts in the New Openness, mostly in Europe, I have explored some ideas here for increased openness in US laboratory animal use. I have envisioned going beyond communicator-controlled decisions on what to show and tell the public about animals, and beyond the Concordat’s sensible invitation for protectionist input on what information to give to the public, or how to allow the public to ask questions, when the result remains competition for the public’s ears, eyes, and sympathies (Figure 1 and Figure 2). I am calling here for invitational openness, or rather, for a renewed commitment to invitational openness, for some of these efforts date backs for decades and some of these efforts are ongoing in the US and other countries. With invitational openness, protectionists and scientists can work together to produce the facts and practices of laboratory animal care and use, and agree at least, in part, to some of the messages the public receives (Figure 3). Can we agree on at least some of the facts, even if interpretations of those facts’ significance, and normative conclusions on what practices to follow?

## Figures and Tables

**Table 1:** Some potential sources of information on animal research information.

**Within the institution**
Senior research scientists
Junior research scientist trainees and technicians
Junior animal care support staff: Animal care and vet techs
Senior animal care support staff: Veterinarians and senior professionals
Institutional management
Public relations and press officers,
Tour leaders, classroom presenters
Public records officers
Compliance staff, annual reports
Whistleblowers
**Outside the institution**
Animal Activists
Animal Protectionists
Journalists
Inspectors writing reports

**Table 2:** Some potential recipients of animal research information.

**Outside the institution**
General public
Scientists
Journalists
Animal activists/protectionists
Tour groups and classes
Inspectors, regulators, accreditation agencies
Lawmakers
Public records requestors
Donors
Patient advocacy groups
**Within the institution**
Institutional non-animal staff
Faculty scholars (including scientists who do not use animals)
Doctors and allied clinical practitioners
Students
Ethics committee members
